# Early Real-World Data to Assess Benefits and Risks of COVID-19 Vaccines: A Systematic Review of Methods

**DOI:** 10.3390/vaccines10111896

**Published:** 2022-11-10

**Authors:** Tatiane B. Ribeiro, Fátima Roque, Fidelia Ida, Ana I. Plácido, Mai Vu, Jose J. Hernández-Muñoz, Maria Teresa Herdeiro

**Affiliations:** 1 Postgraduate Program of Epidemiology, Department of Epidemiology, School of Public Health, Universidade de São Paulo, São Paulo 01246-904, SP, Brazil; 2 Research Unit for Inland Development, Polytechnic of Guarda (UDI/IPG), 6300-559 Guarda, Portugal; 3 CSL Behring, 1020 1st Ave, King of Prussia, PA 19406, USA; 4 Faculty of Pharmacy, HUTECH University, Ho Chi Minh City 70000, Vietnam; 5 School of Pharmacy, University of Eastern Finland, 70210 Kuopio, Finland; 6 Department of Pharmaceutical Sciences, Irma Lerma Rangel College of Pharmacy, Texas A&M University, College Station, TX 77843-0000, USA; 7 Department of Medical Sciences, iBiMED–Institute of Biomedicine, Universidade de Aveiro, 3810-193 Aveiro, Portugal

**Keywords:** COVID-19, real-world data, effectiveness, safety, methods

## Abstract

Since the authorization of the first COVID-19 vaccines in December 2020, multiple studies using real-world data (RWD) have been published to assess their effectiveness/safety profile. This systematic review aimed to characterize the methods and outcomes of studies using RWD for assessment of COVID-19 vaccines, four months after vaccine approval. MEDLINE and EMBASE were searched to identify published studies until 6 May 2021. Two independent researchers selected relevant publications and extracted data from included studies. The risk of bias was assessed using New-Castle Ottawa tools. After screening 1086 studies, 15 were included. Out of the 15 studies, 12 (80%) followed a cohort design, 8 (53%) were based on USA data, 7 (47%) assessed health care professionals, and 14 articles (93%) assessed the BNT162b2 vaccine. Data sources included institutional databases, electronic health records, and patient-generated data. The primary endpoint mainly described was SARS-CoV-2-infection. Hospitalization and mortality were assessed in 2 studies. For the comparability domain, six studies (40%) had a high risk of bias. A few months after the beginning of COVID-19 vaccination, Real-world Evidence (RWE) provided timely safety surveillance and comparative effectiveness with findings that showed similar findings to Randomized control trial (RCT). Most of the initiatives assessed BNT162b2 and were conducted in the USA and used healthcare workers’ data.

## 1. Introduction

The SARS-CoV-2 infection, COVID-19, was first confirmed in humans on 31 December 2019, in China. It rapidly became a worldwide healthcare concern and was declared a pandemic by WHO in March 2020 [1]. Since the beginning of the pandemic, real-world data (RWD) were used to assess the natural history of the disease by case series, evaluate complications arising from the infection, and estimate COVID-19 medication effectiveness. More recently, RWD has been used to assess COVID-19 vaccine effectiveness. The FDA defines RWD as “data relating to patient health status and/or the delivery of healthcare that comes from a number of sources, such as electronic health records, claims and billing activities, and product/disease registries” [2]. The source information of RWD studies are outside the scope of Randomized control trial (RCT) sources. RCT uses stringent eligible criteria, specific populations, and constant monitoring to obtain high internal validity and guarantee that the study protocol is followed. The data obtained from these studies are used for regulatory approval of new drugs. However, clinicians have some concerns that the generalizability of RCT data is not enough [3].

BNT162b2 vaccine received temporary emergency use authorization in the U.K. in December 2020 and, subsequently, several approvals for emergency use in Bahrain, Canada, Mexico, Saudi Arabia, and the USA [4,5]. Published clinical trials showed efficacy ranging from 94% [6] to 95% [7]. As soon as these vaccines were approved for emergency use, several countries started mass vaccination campaigns [8], and RWD began to be generated. By 2 March 2021, more than 247.8 million people have been vaccinated with different vaccines manufactured with diverse technologies in record time between development and current patient use [9].

No study has appraised the current outcomes of COVID-19 studies using RWD to serve as a comprehensive resource for supporting evidence-based practice. This systematic review aimed to characterize the methods and outcomes of studies using RWD to rapidly assess COVID-19 vaccines’ effectiveness and safety.

## 2. Materials and Methods

This study was reported according to the Preferred Reporting Items for Systematic Reviews and Meta-analysis (PRISMA) statement [10]. The protocol was registered on the PROSPERO International Prospective Register of Systematic Reviews under the number: CRD42021252412.

### 2.1. Information Source

A comprehensive search was performed in the most popular databases in the medical field, MEDLINE (Via PubMed) and EMBASE from inception until 6 May 2021. The search strategy combined the population, “COVID-19” and the intervention “Vaccines” as MeSH Terms with respective synonyms as keywords (see Supplementary Material). The search also identified specific observational study designs (Cohort, Case-control, and Cross-sectional), and pragmatic trials.

### 2.2. Eligibility Criteria

We included published studies that used RWD which was defined by the FDA as “data relating to patient health status and/or the delivery of healthcare that comes from a number of sources” [2]. Studies were included if they assessed the effectiveness and safety of COVID-19 vaccines using observational studies (cohort, case-control, or cross-sectional) or pragmatic trials [11]. We excluded studies that included data obtained prior to the beginning of the study. Studies collecting blood samples with prospective intervention were not considered real-world because it was not routine care. We excluded opinion articles, editorials, letters, reviews, and systematic reviews (with or without meta-analysis); exploratory clinical trials or randomized trials that do not mention “pragmatic trial” in the title and/or abstract, and preprints and papers not published under peer review. We restricted the publication language to English, Spanish, or Portuguese.

### 2.3. Study Selection

All records obtained via electronic search were imported into Mendeley^®^ software to remove duplicates. Two researchers (MTH and FR) worked independently using Rayyan^®^ to screen all potential papers by title and abstract. Full-text evaluation was performed independently by three researcher pairs (FI, FR, JJHM, MTH, MV, or TBR). Disagreements were resolved by discussion or, when necessary, by a third reviewer.

### 2.4. Data Collection

For each eligible trial, pairs of researchers (FI, FR, JJHM, MTH, MV, TBR) extracted data independently using a standardized, pilot-tested data extraction form. Reviewers collected information on study characteristics, study design (as reported by the authors), characteristics of the population, methods used to include participants, setting, intervention/exposure, comparator, sample size, country, patients’ enrolment, primary outcome, definition of the main outcome, first three secondary outcomes mentioned, data source, description of methods used for control confounding, and measure of effect from the primary outcome. Reviewers resolved discrepancies by discussion and, when necessary, with adjudication by a third reviewer.

### 2.5. Risk of Bias of Included Studies

The risk of bias was assessed according to each type of study. Observational studies were assessed with New-Castle Ottawa (NOS) [12]. Three NOS adaptations were used according to study design (cohort [12], case-control [13], and cross-sectional studies [14], and the scores ranged from 0 to 9, where the highest score of 9 meant a low risk of bias study. For example, for NOS Cohort, the domain ‘Selection’ included four questions, the highest score for this domain was 4. In the ‘Comparability’ domain, the highest score was 2. For the ‘Outcomes’ domain, the highest score was 3 points.

### 2.6. Data Synthesis

Extracted data were quantitively synthesized and a summary reported on the studies’ population, country, study design, vaccines used, the primary outcome (with details if provided, e.g., polymerase chain reaction (PCR) mentioned in SARS-CoV-2 tests), data source, and primary outcome measures (effectiveness or safety as primary outcome). In addition, methodological aspects related to patient selection and control for confounding were assessed. Effectiveness and safety outcomes were reported separately. The proportions were presented in tables to identify the most frequent characteristics of these studies.

The risk of bias was presented as a figure that grouped the major NOS domains: selection, comparability, and outcomes from all NOS adaptations.

## 3. Results

**Figure 1:**
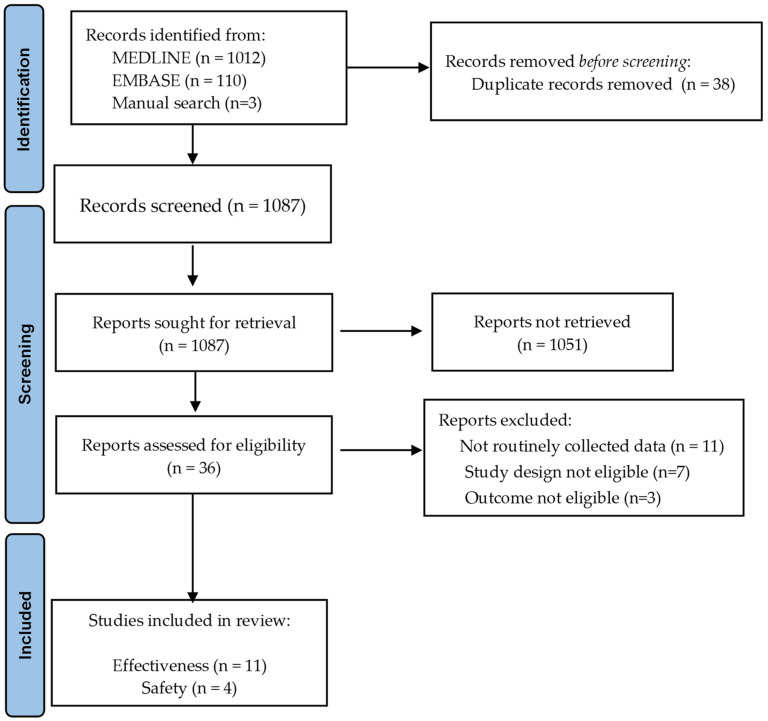
Studies selection flowchart.

After screening 1086 titles and abstracts, 15 studies [15,16,17,18,19,20,21,22,23,24,25,26,27,28,29] using RWD to assess COVID-19 vaccines’ effectiveness and/or safety were identified (Figure 1). A table of excluded full texts is provided in the Supplementary Material (Table S1).

From the included publications which assessed the effectiveness or safety of COVID-19 vaccines using real-world data, 47% (7 of 15 studies) were conducted among health care professionals, 20% (3 of 15 studies) nursing home residents or patients in a hospital setting or other, [15,23,25] and 2 studies, 13% used a nationwide database from Israel and the U.K. [19,22] (Table 1). More than half of the initiatives (53%, 8 of 15 studies) were based in the USA, a fifth of studies were conducted in Israel (20%, 3 of 15 studies), and the others were from UK, Spain, and the Czech Republic database. Majority of the studies (93%, 14 of 15 studies) investigated the use of BNT162b2 and the evaluation was commonly associated with other vaccines such as mRNA-1273 [40% (6 of 15 studies)], and Oxford ChAdOx1 in one study comparing BNT162b2 or ChAdOx1 nCoV-19 (Table 1). Institutional databases (such as human resources in a hospital setting) and specific databases (such as Staff from publicly funded hospitals in the UK, Chicago Department of Public Health) were the most common data source, taking a third of those studies (Table 1).

Electronic health records and patient-generated data, with questionnaires, were reported in each 27% of the studies (Table 1, Table 2 and Table 3). The SARS-CoV2 infection was the most popular primary endpoint in 10 out of 11 studies (92%) (Table 1). Whilst cases were confirmed by PCR in most studies, two studies reporting COVID-19 cases did not clarify the test used (Table 2). Hospitalization and mortality were assessed in two studies each (18%) (Table 2). Vaccine effectiveness from the studies was reported in Table 2.

Three studies assessed safety (adverse events information) as a primary. One study also assessed reactogenicity, and another reported acute allergic reactions in different nCoV-19 vaccines. The most common adverse reactions reported were injection site pain, fatigue, headache, and muscle pain (Table 3). Cohort studies were the most frequent study design, reported by the authors, 12 of 15 studies (80%), studies assessing safety were frequently cross-sectional studies (2 in 15 studies, 13%), while the only study that assessed effectiveness was a case-control study. A pragmatic trial was not recorded during the time we assessed publication databases (Table 1).

**Figure 2:**
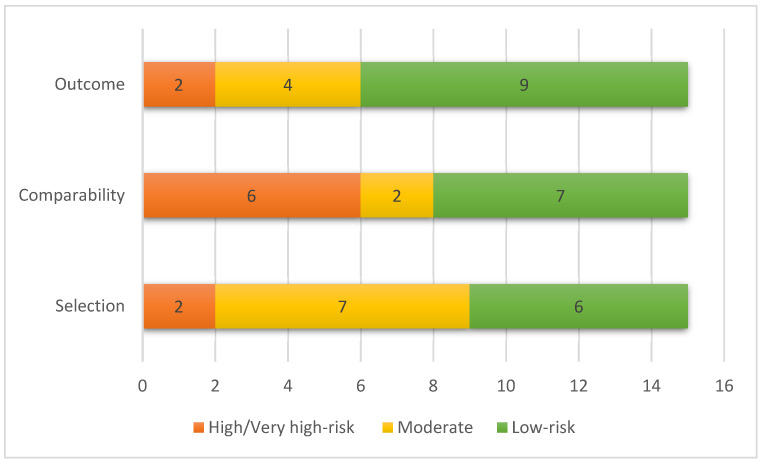
Pooled risk of bias of studies, according to New-Castle Ottawa main domains.

A risk of bias was assessed using the NOS tool. About 38% of the studies presented a moderate risk of bias, 22% had a high risk of bias, and 40% had a low risk of bias. For ‘Outcome’ related questions, 60% of the studies presented a low risk of bias, and 22% had a high risk of bias. For ‘Comparability’, 47% of the studies showed a low risk of bias and 40% had an increased risk of bias (Figure 2). Among the studies with low risk of bias, different methods were used to control for confounding variables, including (Supplementary Material, Table S2): Poisson regression (two studies), Mixed-effect model (two studies), Linear regression (one study), Binomial regression (one study), Logistic regression (one study), and Propensity score-matching (one study). The papers generally adjusted by different variables, the most common being sex, age, and ethnicity. Furthermore, each study was adjusted by the variables related to specific settings (e.g., occupation for studies carried out among hospital professionals, patient residence relative to the hospital (local vs. nonlocal) for nursing/community residencies, or risk exposure), Supplementary Material (Table S2).

## 4. Discussion

Our systematic review of methods showed that four months after COVID-19 vaccines approval, fifteen original studies using RWD were published to assess the effectiveness and safety of vaccines as the primary source of evidence critical for supporting medical and public health decisions [30].

Studies using RWD are important to present the outcomes in the real world, such as effectiveness and safety, beyond explanatory RCT, which often employs specific populations in specialized environments, to control for variability, and to ensure data quality [11]. Data included from low risk of bias studies with COVID-19 vaccines showed that the effectiveness in the real world was similar to that reported in RCT, ranging from 94% [5] to 95% [7]. One low-risk of bias study came from a nationwide database, likewise the first two initiatives used nationwide (Israel and Scotland) information about vaccination. This rapid response using RWD and subsequent publication of high-quality papers reflected the well-organized international databases of these healthcare systems, which might be an example for other countries for on-time health care data generation [31,32,33].

The characteristics seen in studies included in this review pointed out the historical facts beyond the COVID-19 vaccination campaigns. BNT162b2 from BioNTech–Pfizer received a temporary emergency use authorization from the U.K. Health care Agency in early December 2020 and, subsequently, several authorizations for emergency use in Bahrain, Canada, Mexico, Saudi Arabia, and the USA [4]. When the first vaccine entered the market for emergency use, some countries began a mass vaccination campaign, starting with the health care professionals working in hospitals [8]. This study which assessed these initial initiatives (published on or before 6 May 2020), found that countries that published more papers using RWD started the vaccination campaign first, such as USA and Israel. More than half of the studies found in this review used USA health care workers’ data. Due to the volume of papers published in the USA, the vaccines approved in this country during the period were the most studied, as this study shows. The BNT162b2 was investigated in almost all studies, alone or compared to other vaccines (mRNA-1273).

Researchers might have to be cautious about the inclusion of RWD. As per definition, it is unclear if all information published after RCT, using population data, might be included as RWD. By reassessing FDA definition and literature [9,10], we included studies that used data routinely collected. Thus, studies designed to actively collect samples, such as blood samples to assess immunogenicity in vaccinated people, were not included. This discussion is pertinent to the scientific community’s understanding of the RWE definition and to inform future systematic reviews that might include this type of study.

Some authors also argue about the misuse of the term ‘RWE’, as this term might confuse readers and promote the misinterpretation of reporting guidelines [34]. Studies using RWD should follow a very rigid reporting according to its design [35], as suggested by the reporting guidelines listed in the EQUATOR initiative [36]. RWE can be an observational study (case series, cross-sectional, case-control, cohort) or pragmatic trial, and it is dependent on whether the data came from routine care. In this systematic review, no pragmatic trial was found, and all observational studies were assessed for methodological quality according to study design using different New Castle Ottawa adaptations [12,13,14]. We did not find studies that employed the self-controlled case series (SCCS) which is an epidemiological method for which a person acts as their own control that was developed for the evaluation of vaccine safety, and has been applied in settings where the exact information of the size of population risk is unavailable, or an appropriate comparison group is difficult to find [30].

The included studies were heterogeneous, with the majority having a moderate or high risk of bias. Another major problem seen in these papers was from the comparability domain, related to controlling confounding that is critical for precise and unbiased estimation in observation studies [37]. Caution should be applied in assessing analytical methods to control for confounding in the critical appraisal of studies using RWD. This study showed that several studies did not control for confounding, and the few which did applied different methods for covariates adjustments. Even studies with large sample sizes might be biased and have methodological issues related to patient selection, outcome measurement and reporting, and results should be cautiously interpreted. This study did not assess the useability of the data sources and whether the study designs avoided major biases.

## 5. Conclusions

Our systematic methodological review showed that a few months after COVID-19 vaccines approval, fifteen original studies with RWD were published to assess the effectiveness and safety of the vaccines, and their results were similar to evidence from RCTs. Most of the initiatives assessed BNT162b2, emanated from the USA, and used healthcare workers’ data.

Observational studies with RWD conducted, following good research practices proposed in reporting guidelines, decrease bias and provide confident results estimations. Previously structured databases might be valuable to the fast generation of important public health data.

## Figures and Tables

**Table 1:** General characteristics of studies using real-world data to assess effectiveness or safety in the first 4 months after vaccines’ first approval.

Characteristics	Number	Proportion from the Total of Studies
Population (*n* = 15)		
Healthcare professionals	7 of 15	47%
Nursing home residents	3 of 15	20%
Patient in hospital setting or Other setting	3 of 15	20%
Population-based	2 of 15	13%
Country (*n* = 15)		
USA	8 of 15	53%
Israel	3 of 15	20%
UK	2 of 15	13%
Spain	1 of 15	7%
Czech Republic	1 of 15	7%
Study type (*n* = 15)		
Cohort	12 of 15	80%
Cross-sectional ¹	2 of 15	13%
Case-control	1 of 15	7%
Vaccines (study might include >1 vaccine)		
BNT162b2	14 of 15	93%
mRNA-1273	6 of 15	40%
ChAdOx1	1 of 15	7%
Non-specified	1 of 15	7%
Data source (study might include >1 database)		
Specific database ²	5 of 15	33%
Institutional database	5 of 15	33%
Eletronic-Health records	4 of 15	27%
Patient-generated data	4 of 15	27%
Effectiveness primary outcome (study might include >1 outcome)		
SARS-CoV-2 infection	10 of 11	91%
Hospitalization	2 of 11	18%
Mortality	2 of 11	18%
Safety Outcomes (study might include >1 outcome)		
Adverse events general	3 of 4	75%
Acute allergic reaction	1 of 4	25%
Reactogenicity	1 of 4	25%

^1^ Only safety studies. ^2^ Specific database: SIREN database (Staff from publicly funded hospitals in the UK), Chicago Department of Public Health (CDPH) database, HERO database (USA eight locations), hospital setting, Early Pandemic Evaluation and Enhanced Surveillance of COVID-19—EAVE II Electronic Communication of Surveillance in Scotland (ECOSS) Turas Vaccination Management Tool (TVMT), CMS National Health Safety Network (NHSN).

**Table 2:** Characteristics of studies included assessing the effectiveness of COVID-19 vaccines.

Study	Population N (Sample Size)	Country	COVID-19 Vaccine	Study Design	Database	Main Outcomes	Result (Relative Measure Compared to Unvaccinated) Effectiveness (%), If Available
Hall et al., 2021 [15]	Healthcare workers, and staff from hospital *n* = 23,324	UK	BNT162b2	Cohort	SIREN database (Staff from publicly funded hospitals in the UK)	SARS-CoV-2 infection confirmed by a PCR test	OR 0.59 [CI 95% 0.54–0.64) 85% (CI 95% 74–96)
Rudolph et al., 2021 [16]	Community living center residents N = 130 clinics (>6000 residents)	USA and Puerto Rico	Not specified	Cohort	COVID nursing home data website and electronic health records	Positive SARS-CoV-2 tests as reported	RR 0.37 (CI 95% 0.20–0.68)
Swift et al., 2021 [17]	Healthcare workers *n* = 71,152	USA	BNT162b2 or mRNA-1273	Cohort	Occupational Health Services database	SARS-CoV-2 infection confirmed by a PCR test	BNT162b2r % effectiveness 2 doses = 0.968 (0.953, 0.978); mRNA-1273a % effectiveness 2 doses = 0.986 (0.901, 0.998) BNT162b2 % effectiveness 1 dose = 0.781 (0.711, 0.820) mRNA-1273 % effectiveness 1 dose = 0.912 (0.806, 0.961)
Tande et al., 2021 [18]	Patients screened (preprocedural and presurgical) in clinical/hospital *n* = 48,333	USA	BNT162b2 or mRNA-1273	Cohort	Eletronic Health records and institutionally curated COVID-19 database	SARS-CoV-2 infection confirmed by a PCR test	RR 0.35 (CI 95% 0.26–0.47)
Gras-Valentí et al., 2021 [19]	Healthcare workers, and staff from hospitals and clinics *n* = 268	Spanish	BNT162b2	Case-Control	Hospital workforce database	SARS-CoV-2 infection confirmed by a PCR test	OR 0.47 (0.23–0.99)
Teran et al., 2021 [20]	Nursing Facility Residents and Staff Members *n* = 627	USA	BNT162b2 or mRNA-1273	Cohort	Chicago Department of Public Health (CDPH) database	SARS-CoV-2 infection (NE detection)	22 of 627 SARS-CoV-2 infections occurred among vaccinated
Thompson et al., 2021 [21]	Healthcare workers, and staff from hospital *n* = 3950	USA	BNT162b2 or mRNA-1273	Cohort	HERO database (USA eight locations), hospital setting	SARS-CoV-2 infection confirmed by a PCR test	Fully immunized 90% (68–97) Partially immunized 80% (59–90)
Vasileiou et al., 2021 [22]	Scotland population-based *n* = 1,331,993 (vaccinated)	Scotland	BNT162b2 or ChAdOx1 nCoV-19	Cohort	Early Pandemic Evaluation and Enhanced Surveillance of COVID-19—EAVE II Electronic Communication of Surveillance in Scotland (ECOSS) Turas Vaccination Management Tool (TVMT)	Hospital admissions with COVID-19 as the main cause of admission	ChAdOx1 vaccine 88% (95% CI 75–94) BNT162b2 mRNA 91% (95% CI 85–94)
Angel et al., 2021 [23]	Health care workers from hospital *n* = 6710	Israel	BNT162b2	Cohort	Hospital workforce database	Symptomatic SARS-CoV-2 infection confirmed by a PCR test	Adjusted IRR 0.14 [95% CI, 0.07–0.31)
Domi et al., 2021 [24]	Nursing Facility Residents and staffs *n* = 2501	USA	BNT162b2	Cohort	CMS National Health Safety Network (NHSN) Public File data	New COVID-19 resident cases per resident-week and Resident deaths	Resident cases (6w) IRR: 0.64 (95% CI 0.48–0.86) Resident deaths (6w) IRR: 0.45 (95%CI 0.31–0.65)
Dagan et al., 2021 [25]	Israel population-based *n* = 596,618 (vaccinated)	Israel	BNT162b2	Cohort	Electronic medical records of Clalit Health Services (CHS)	SARS-CoV-2 infection confirmed by a PCR test Hospital admission for COVID-19 Death from COVID-19	7 or more after 2nd dose Prevent Infection: 92% (95% CI, 88 to 95) Prevent hospitalization: 87% (95% CI, 55 to 100) Prevent Death: 72% (95% CI, 19 to 100)

Legend: BNT162b2 (Pfizer vaccine), ChAdOx1 nCoV-19 (Oxford–AstraZeneca vaccine), IRR: incidence rate ratio, mRNA-1273 (Moderna vaccine), OR: Odds Ratio, RR: relative risk, Rra: adjusted relative risk, USA: United States, UK: United Kingdom.

**Table 3:** Characteristics of studies included assessing the safety of COVID-19 vaccines.

Study	Population	Country	COVID-19 Vaccine	Study Design	Database	Main Outcome	Result
Ou et al., 2021 [26]	Solid organ transplant recipients	USA	BNT162b2 or mRNA-1273	Cohort	Patient-generated data by questionnaires from Social media or transplant centers (Johns Hopkins)	Reactogenicity and most frequent adverse events	The most common were pain, fatigue (Dose1–36%; Dose2–56%), and headache (D1–28%; D2–42%)
Riad et al., 2021 [27]	Health care workers	Czech Republic	BNT162b2	Cross-Sectional	Patient-generated data by questionnaire, from hospital setting	Prevalence of adverse effects	Injection site pain (89.8%), fatigue (62.2%), headache (45.6%), muscle pain (37.1%), and chills (33.9%)
Achiron et al., 2021 [28]	Multiple sclerosis patients	Israel	BNT162b2	Cohort	Patient-generated data from Multiple Sclerosis center	Adverse event proportion	Safety profile of COVID-19 vaccine was characterized by pain at the injection site (14.2%), fatigue (15.9%), and headache (7.3%)
Blumenthal et al., 2021 [29]	Health care workers	USA	BNT162b2 or mRNA-1273	Cross-Sectional	Eletronic health records and patient-generated data “self-reported” from hospitals	Acute Allergic Reactions	Acute allergic reactions were reported by 1365 employees overall (2.10% [95% CI, 1.99–2.22%]), more frequently with the Moderna vaccine compared with Pfizer-BioNTech (2.20% [95% CI, 2.06–2.35%] vs. 1.95% [95% CI, 1.79–2.13%]; P = 0.03)

Legend: BNT162b2 (Pfizer vaccine), ChAdOx1 nCoV-19 (Oxford–AstraZeneca vaccine), mRNA-1273 (Moderna vaccine), USA: United States.

## Data Availability

Not applicable.

## Supplementary Materials

The following supporting information can be downloaded at: https://www.mdpi.com/article/10.3390/vaccines10111896/s1, Table S1: Excluded studies after full text reading and reasons; Table S2: Methodological characteristics of COVID-19 studies using real-world data for effectiveness.
