# Supplementary material for: Early Real-World Data to Assess Benefits and Risks of COVID-19 Vaccines: A Systematic Review of Methods
Source: Vaccines (Basel). 2022 Nov 10;10(11):1896. doi: 10.3390/vaccines10111896 (PMC9697784; doi:10.3390/vaccines10111896)
Supplement: Supplementary file 1 [file vaccines-10-01896-s001.zip › vaccines-2000289-supplementary.pdf]

Supplementary material

Table S1: Excluded studies after full text reading and reasons

First author (last name)	Year of publication	DOI	
Frantzen	2021	10.1093/ndt/gfab165	Not routinelly collected data
Geisen	2021	10.1136/annrheumdis-2021-220272	Not routinelly collected data
Hacisuleyman	2021	10.1056/NEJMoa2105000	Not routinelly collected data
Kalimuddin	2021	10.1016/j.medj.2021.04.003	Not routinelly collected data
Monin	2021	10.1016/S1470-2045(21)00213-8	Not routinelly collected data
Muller	2021	10.1093/cid/ciab381	Not routinelly collected data
Remmel	2021	10.1038/d41586-021-00290-x	Study design didnt met elegibility criteria
Rozen-Zvi	2021	10.1016/j.cmi.2021.04.028	Not routinelly collected data
Bernstine	2021	10.1097/RLU.0000000000003648	Outcome did not met elegibility criteria
Wang	2021	10.1038/s41586-021-03324-6	Not routinelly collected data
Zhou	2021	10.1016/j.cell.2021.02.037	Not routinelly collected data
Dean	2021	10.1016/S0140-6736(21)00765-	Study design didnt met elegibility criteria
Ebinger	2021	10.1038/s41591-021-01325-6	Not routinelly collected data
Gray	2021	10.1016/j.ajog.2021.03.023	Not routinelly collected data
Benenson	2021	10.1056/NEJMc2101951	Study design didnt met elegibility criteria
Munitz	2021	NA	Outcome did not met elegibility criteria
Gharpure	2021	10.15585/mmwr.mm7005e2	Outcome did not met elegibility criteria
Lillie	2021	10.1093/cid/ciab351	Study design didnt met elegibility criteria
Wolf	2021	10.3390/jcm10081599	Study design didnt met elegibility criteria
Xu	2021	10.1097/RLU.0000000000003597	Study design didnt met elegibility criteria
Jedlowski	2021	13030/qt4xs486zg.	Study design didnt met elegibility criteria

Table S2: Methodological characteristics of Covid-19 studies using real-world data for effectiveness.

Study	Methods to select participants	Method to assess primary outcome	Method to control for confounding	Study quality NOS
**Hall et al., 2021**	SIREN database - Asymptomatic PCR testing every 14-d and monthly antibody testing	"SARS-CoV-2 infection confirmed by a PCR test, defined as a new PCR positive result during follow-up for the negative cohort and a reinfection during the follow-up in the positive cohort, irrespective of symptom status."	"Mixed-effect multi-variable, hazard mixed-effects model (shared frailty-type model) using a Poisson distribution"	9 of 9
**Rudolph et al., 2021**	Community Living Center (CLC) residents included in Veterans Affairs electronic records	"COVID-19 Nursing Home Data public website and reported Covid-19 infection."	NA	4 of 9
**Swift et al., 2021**	Healthcare professionals from MayoClinics 76,000 personnel	"All actively employed HCP with a positive molecular assay for SARS-CoV-2 are contacted by Occupational Health Services (OHS) for systematic index case interview and contact tracing as described elsewhere (8). Test results, exposure investigation details, vaccination status, and employee demographics are included in the OHS record. Diagnostic molecular assays are conducted for any new COVID-19 symptom. In addition, asymptomatic HCP are tested serially for long-term care facility surveillance, for 2 weeks following COVID-19 exposure at work or in the community, and a variety of other reasons such as anticipated personal travel or medical procedures."	"We used linear regression modal with robust standard error in with we assumed the Poisson distribution… Adjusting for age, gender, region, job, and week of vaccination, VE for partial and complete vaccination."	8 of 9
**Tande et al., 2021**	All consecutive molecular screening tests performed in adult (≥18 years old)	"Molecular testing was performed through a combination of emergency use authorized methods	"All analyses were repeated with adjustment in mixed-effects models with random intercepts for each Mayo Clinic site	7 of 9

	patients at Mayo Clinic campuses, underwent preprocedural and presurgical SARS-CoV-2 molecular testing	depending on the Mayo Clinic location, including a SARS-CoV-2 laboratory-developed real-time polymerase chain reaction (PCR) [10], the APTIMA SARSCoV-2 transcription-mediated amplification assay (Hologic, Marlborough, MA), and the Abbott RealTime SARS-CoV-2 real-time PCR method.”	(Rochester, Mayo Clinic Health System, and Arizona), a random residual to correct for intrapatient repeated measures, and fixed effects for age, sex, race/ethnicity, and patient residence relative to the hospital (local vs. nonlocal).”	
**Gras-Valentí et al., 2021**	Health care workers contractually linked to the Alicante-Hospital General Health Department (consisting of a tertiary hospital, twelve primary care centers, and 5,345 SPs) were included	PCR for the determination of SARS-CoV-2 in a nasopharyngeal aspirate sample.	“En el modelo de regresión logística se introdujeron aquellas variables que mostraron diferencias estadísticamente significativas entre los grupos de PS vacunados y no vacunados, así como aquellas que se asociaron de forma significativa al desarrollo de la infección por SARS-CoV-2.”	6 of 9
**Teran et al., 2021**	Nursing Facility Residents and Staff Members from Chicago, Illinois with identified a SARS-CoV-2 infection	Routine screening, the Chicago Department of Public Health (CDPH) identified a SARS-CoV-2 infection in a SNF resident >14 days after receiving the second dose of a two-dose COVID-19 vaccination series.	NA	7 of 9
**Thompson et al., 2021**	Health Care Personnel, First Responders, and Other Essential and Frontline Workers from HEROES-RECOVER, a network of longitudinal cohorts in eight locations (Phoenix, Tucson, and other areas in Arizona; Miami, Florida; Duluth, Minnesota; Portland, Oregon; Temple, Texas; and Salt Lake City, Utah)	“Participants self-collected a midturbinate nasal swab weekly, regardless of COVID-19–associated illness symptom status, and collected an additional nasal swab and saliva specimen at the onset of COVID-19–associated illness. Specimens shipped on cold packs were tested by RT-PCR assay at Marshfield Clinic Laboratory (Marshfield, Wisconsin) to determine SARS-CoV-2 infections (PCR-confirmed infection).”	Mentioned a "sensitivity analyses," inclusion of other covariates (sex, age, ethnicity, and occupation)	7 of 9

**Vasileiou et al., 2021**	Scotland databases with nationwide information	“Laboratory data from ECOSS included all rtPCR test results from both National Health Service laboratories (Pillar 1) and Lighthouse Government laboratories.”	“Poisson regression adjusting for an offset representing the time at risk and time-dependent Cox models (considering the time at risk) were used to derive the rate ratios, hazard ratios, and 95% CIs for the association of vaccination with COVID-19 hospital admissions. Cox models included spline terms for age and number of rtPCR tests before vaccination (a marker for healthcare workers, social care workers, and care home residents who had repeated tests). Adjusted for time (in weeks), age, sex, Scottish Index of Multiple Deprivation, number of rtPCR tests before vaccination, number of underlying medical conditions, and inverse propensity of being vaccinated. - Any effects observed in less than 14 days are mainly due to vaccine programme effects.”	8 of 9
**Angel et al., 2021**	Health care workers who received at least 1 vaccine dose between December 20th, 2020, and February 25th, 2021, were assigned to the vaccinated group. The study was conducted at the Tel Aviv Sourasky Medical Center, a tertiary medical center that employs approximately 7500 health care workers and close to 4000 nonsalaried health care workers (e.g., students, volunteers)	“The PCR tests anD symptoms were performed according to the screening policy of the hospital. The screening policy changed during the course of the study period as follows: from December 20th, 2020, to January 02nd, 2021 (period 1), health care workers were screened monthly or biweekly depending on their risk of SARS-CoV-2 exposure; from January 03rd to 14, 2021 (period 2), hospital-wide screening was done, including all health care workers regardless of vaccination status; and from January 15th,	“Multivariable Poisson regression model. he adjusted IRR was estimated using a multivariable Poisson regression model with confirmed cases as a response variable and group assignment (vaccinated vs. unvaccinated), age, sex, employment sector, exposure risk, and number of PCR tests for each health care worker in the time frame under observation as explanatory variables. To account for possible differences between the groups, including the possibility of a detection bias due to a difference in the number of tests carried out per group, a	7 of 9

		2021-onward (period 3), health care workers with medium to high exposure risk (defined below) and non–fully vaccinated health care workers were screened monthly to weekly in accordance with evolving hospital directives (Figure 1A and eMethods in the Supplement). The primary outcome was incidence of SARS-CoV-2 infection among fully vaccinated health care workers compared with unvaccinated health care workers who did not test positive for SARS-CoV-2 infection within the first 28 days of follow-up."	propensity score-adjusted sensitivity analysis11 was performed"	
**Domi et al., 2021**	All Nursing Homes who held a vaccine clinic from the first 17 states to initiate clinics as part of the Pharmacy Partnership for Long-Term Care Program.	"The NHSN Public File contains weekly counts of incident staff and resident COVID-19 cases and deaths reported by LTC facilities to the CDC NHSN system."	"The negative binomial model addresses the issue of overdispersion by including a dispersion parameter that relaxes the assumption of equal mean and variance of the Poisson model. PLUS adjust for Calendar week; county 7-day average rate of COVID-19; urban location; certified beds divided into low (≤50 beds), medium 51–120 beds (referent group), and large (>120 beds); racial minority census by quartile with the lowest group serving as the referent group; Hispanic non-white census by quartile with the lowest group serving as the referent group; and the level of registered nurses divided into low (<0.499 HPRD), medium (0.499–0.987 HPRD, referent group), and high (>0.987 HPRD), all covariates previously shown to be associated with COVID-19 cases."	6 of 9
**Dagan et al., 2021**	Data from Clalit Health Services	"SARS-CoV-2 infection without documented	"Covariate balance after matching was evaluated	9 of 9

	(CHS), the largest of four integrated health care organizations in Israel, which insures 4.7 million patients (53% of the population).	symptoms, as an imperfect proxy for asymptomatic infection (since mild symptoms may not be documented).”	with the use of a plot of the mean differences between variable values (standardized for continuous variables) for the vaccinated and unvaccinated groups, with a difference of 0.1 or less considered to be acceptable.”	
**Ou et al., 2021**	Patient-generated data by questionnaires from Social media or transplant centers (Johns Hopkins)	NA	NA	3 of 9
**Riad et al., 2021**	Patient-generated data by questionnaire, from hospital setting	NA	NA	6 of 9
**Achiron et al., 2021**	Patient-generated data from MS center	NA	NA	5 of 9
**Blumenthal et al., 2021**	Eletronic health records And Patient-generated data from self-reported from hospital	NA	NA	6 of 9
